# Rapid Detection and Antimicrobial Susceptibility Testing of Pathogens Using AgNPs-Invertase Complexes and the Personal Glucose Meter

**DOI:** 10.3389/fbioe.2021.795415

**Published:** 2022-01-18

**Authors:** Laibao Zheng, Yunqiu Shen, Wenjia Dong, Chaochuan Zheng, Ruolan Zhou, Yong-Liang Lou

**Affiliations:** Wenzhou Key Laboratory of Sanitary Microbiology, Key Laboratory of Laboratory Medicine, Ministry of Education, School of Laboratory Medicine and Life Science, Wenzhou Medical University, Wenzhou, China

**Keywords:** nanoparticle-enzyme complexes, personal glucose meter, pathogen detection, antimicrobial susceptibility testing, rapid detection

## Abstract

Rapid detection of pathogens and assessment of antimicrobial susceptibility is of great importance for public health, especially in resource-limiting regions. Herein, we developed a rapid, portable, and universal detection method for bacteria using AgNPs-invertase complexes and the personal glucose meter (PGM). In the presence of bacteria, the invertase could be released from AgNPs-invertase complexes where its enzyme activity of invertase was inhibited. Then, the enzyme activity of invertase was restored and could convert sucrose into glucose measured by a commercially PGM. There was a good linear relationship between PGM signal and concentration of *E. coli* or *S. aureus* as the bacteria model with high sensitivity. And our proposed biosensor was proved to be a rapid and reliable method for antimicrobial susceptibility testing within 4 h with consistent results of Minimum Inhibitory Concentrations (MICs) testing, providing a portable and convenient method to treat infected patients with correct antibiotics and reduce the production of antibiotic-resistant bacteria, especially for resource-limiting settings.

## Introduction

Rapid detection of pathogens and assessment of antimicrobial susceptibility is of great importance for the treatment of infectious diseases, especially in resource-limiting regions. The well-established pathogen detection and antimicrobial susceptibility testing (AST) methods, including culture-based methods, PCR, and mass spectrometry, are classical and useful. However, long detection periods, the need for expensive instruments, and professional operation limit their application in point-of-care testing. Moreover, the lack of point-of-care testing methods for pathogens will lead to the outbreak and spread of infectious diseases (Váradi et al., 2017; Xiao et al., 2021). Therefore, rapid and sensitive detection and AST method for pathogens are still in urgent need.

Thanks to the development of nanotechnology, various colorimetric, fluorescent, and electrochemical biosensors have been developed for rapid and sensitive detection of pathogenic bacteria using functionalized nanomaterials, such as gold/silver nanoparticles (Bi et al., 2020), carbon dots (Zheng et al., 2019a), gold/silver nanoclusters (Zheng et al., 2018; Zheng et al., 2019b; Chen et al., 2021), and magnetic nanoparticles (Wang et al., 2020b). These nanomaterials usually recognize and bind to the surface of bacteria cells through recognition elements, including antibodies (Bu et al., 2020; Wang T. et al., 2020), aptamer (Hua et al., 2018; Wang et al., 2020a), antimicrobial peptides (Pranantyo et al., 2019), and positively charged ligands (Berry et al., 2005). Unlike specific recognition elements (e.g., antibody, aptamer), positively charged ligands bind to bacteria non-specifically through electrostatic interaction, providing a versatile detection method for a wide range of bacterial strains (Phillips et al., 2008; Li et al., 2017; Wang et al., 2019). The positive-charged nanoparticles conjugated with enzymes proved to be a useful tool for bacteria detection. Cationic nanoparticles could bind to anionic enzyme through electrostatic interactions resulting in the inhibition of the enzymatic activity. While negative or neutral charged nanoparticles showed no inhibition effect towards the enzyme (Miranda et al., 2011). The surface of bacteria was negatively charged due to phosphate and carboxyl groups and could easily bind to positive-charged surfaces (Phillips et al., 2008; Ahmed et al., 2014). In the presence of bacteria, Cationic nanoparticles could bind to bacteria forming nanoparticle-bacteria conjugates and enzymes would be released from nanoparticle-enzyme complexes, resulting in the recovery of the enzymatic activity. Up to date, a few colorimetric and smell-based rapid bacteria detection methods based on nanoparticle-enzyme (e.g., β-galactosidase (β-Gal) (Miranda et al., 2011; Thiramanas and Laocharoensuk, 2016), lipase (Duncan et al., 2017), and urease (Singh et al., 2019) complexes have been reported. However, these methods are rather qualitative, and the quantitative assay is still requiring advanced instruments such as UV-vis spectrometry.

Recently, personal glucose meters (PGMs), the successful point-of-care diagnostic tools for blood glucose measurement in the medical diagnostic field for decades, are devoted to being repurposed for quantitative detection of a variety of analytical targets other than blood glucose due to they are portable, inexpensive, and quantitative. Since Lu’s group first reported the portable detection of other analytical targets using PGMs, the rapid and quantitative detection of DNA (Xiang and Lu, 2011; Xiang and Lu, 2012), metal ions (Xiang and Lu, 2013; Qiu et al., 2016), and bacteria (Wang et al., 2015; Wan et al., 2016; Yang et al., 2021) using PGMs have been achieved by researchers. The glucose-generating enzyme or glucose combined with recognition elements binds to targets to generate a glucose signal, which is measured using PGMs. Antibody or aptamer as the recognition element-based sandwich biosensor were usually used for bacteria detection in PGM based method. A portable and quantitative immunochromatographic assay was reported for *E. coli* O157:H7 detection with a PGM(Huang et al., 2018). PGM-based method using aptamer as recognition and hybridization chain reaction as signal amplification strategy was developed for portable detection of *S. aureus* (Yang et al., 2021).

Herein, we developed a rapid, portable, and quantitative detection method for bacteria using AgNPs-invertase complexes and PGMs. In the AgNPs-invertase complex system, the enzyme activity of invertase was inhibited by AgNPs electrostatically bound to the invertase. However, the invertase was released and restored its enzyme activity in the presence of bacteria. Then, the active invertase could convert sucrose into glucose measured by a commercially PGM, allowing the one-pot quantitative detection of bacteria without multiple washing and rinsing steps. Our proposed biosensor provides a portable and universal method for bacteria detection. And the proof-of-concept experiments were conducted by measuring the concentration of *E. coli* after being treated with antibiotics.

## Materials and Methods

### Regents and Materials

Invertase from baker’s yeast (*S. cerevisiae*) was purchased from Sigma–Aldrich (St. Louis, MO, United States). Sodium citrate, tannic acid, silver nitrate, polyethyleneimine (M.w. = 1800), sucrose, MES, and other chemicals were of analytical reagent grade from Aladdin Reagent Co. Ltd (Shanghai, China). The glucose level was measured using a commercial PGM (Omnitest plus, B. Braun, Melsungen, Germany). Ultrapure water (18.2 MΩ cm) was used throughout the experiments.

### Synthesis of PEI-AgNPs

The positive charged PEI-AgNPs were prepared according to the previously reported method (Singh et al., 2019). Tannic acid-capped anionic AgNPs were first synthesized by mixing tannic acid (5 mM) and sodium citrate (0.025 mM) into 50 ml water boiled along with vigorous stirring. Then 25 mM silver nitrate was added into the solution with a further reaction for 20 min. The as-prepared tannic acid-capped AgNPs were then washed twice by centrifugation at 10,000 rpm for 40 min at 4°C and adjusted OD_400_ = 1.0. To prepare PEI-AgNPs, 1 ml tannic acid capped anionic AgNPs was added into 1 ml 0.03 mg/ml PEI solution with vigorous stirring for 60 min at room temperature. The PEI-AgNPs were washed twice by centrifugation at 10,000 rpm for 40 min and adjusted to OD_400_ = 1.0 and stored at 4°C for further use.

### Preparation of Bacteria

*Escherichia coli* (*E. coli*) and *Staphylococcus aureus* (*S. aureus*) used in our experiments were cultured in Luria–Bertani (LB) liquid media at 37°C overnight. Then the bacteria suspension was rinsed three times by centrifugation at 4,000 rpm for 5 min and diluted to the desired concentration ranging from 10 to 1.00 × 10^9^ cfu/ml in MES buffer or tap water. The bacteria concentrations were determined using the plate counting method.

### Procedures for Bacteria Detection

AgNPs-invertase complexes were formed by mixing invertase solution (0.8 μg/ml, 30 μL) and the as-prepared PEI-AgNPs (3 μL) for 10 min. Then, 117 μL bacteria of different concentrations or MES buffer were added and incubated for 15 min at room temperature. After adding the sucrose solution (0.5 M, 50 μL), the solution was allowed to react for 20 min at 55°C, followed by measured using the PGM.

### AST of Bacteria

The AST of *E. coli* towards four antibiotics (colistin, spectinomycin, streptomycin, tetracycline) were confirmed using Minimum Inhibitory Concentrations (MICs) testing according to Clinical and Laboratory Standard Institute (Cockerill et al., 2012). Briefly, the suspensions of *E. coli* at a concentration of 0.5 McFarland were diluted 1,000 times with Mueller Hinton Broth and then treated with different concentrations of antibiotics from 0 to 64 μg/ml. For the traditional method, the MIC values are determined by the lowest concentration of antibiotics without visible growth after incubation for 18–24 h at 37°C. Using our proposed method, the bacteria suspension incubated for 5 h was measured to evaluate the antimicrobial susceptibility of *E. coli*.

## Results

### Detection Principle

**FIGURE 1:**
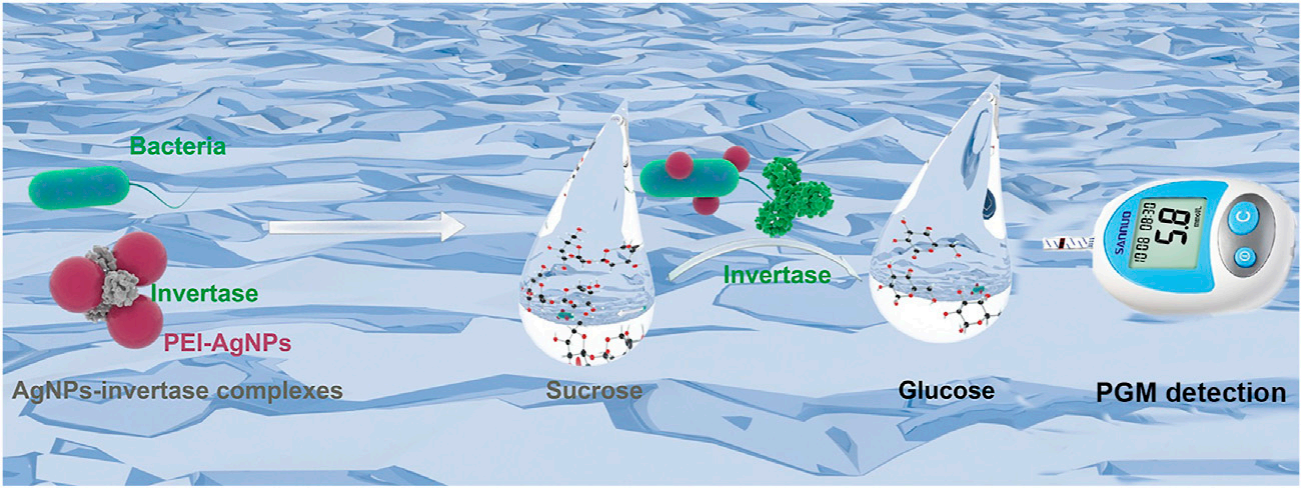
Schematic illustration of the simple, portable, and quantitative detection method for bacteria using AgNPs-invertase complexes and PGMs.

The detection principle of our designed potable and sensitive biosensor for bacteria detection using nanoparticle-enzyme complexes and the PGM is schematically illustrated in Figure 1. The biosensor combines three main components: 1) invertase, an anionic enzyme, to provide signal amplification and convert sucrose into glucose measured by PGMs; 2) sucrose, a catalytic substrate of invertase; 3) PEI-AgNPs, a cationic nanoparticle, which could recognize bacteria and reversibly bind to invertase, inhibiting the catalytic activity. The catalytic activity of invertase is inhibited without denaturation by forming AgNPs-invertase complexes through electrostatic interaction. However, in the presence of bacteria, the cationic AgNPs competitively bind to the anionic surface of bacteria to displace the invertase with the recovery of enzyme activity. The active invertase converts sucrose into glucose, which could be measured by a commercially PGM. Thus, the concentration of bacteria could be quantified using a PGM.

### Feasibility Study

**FIGURE 2:**
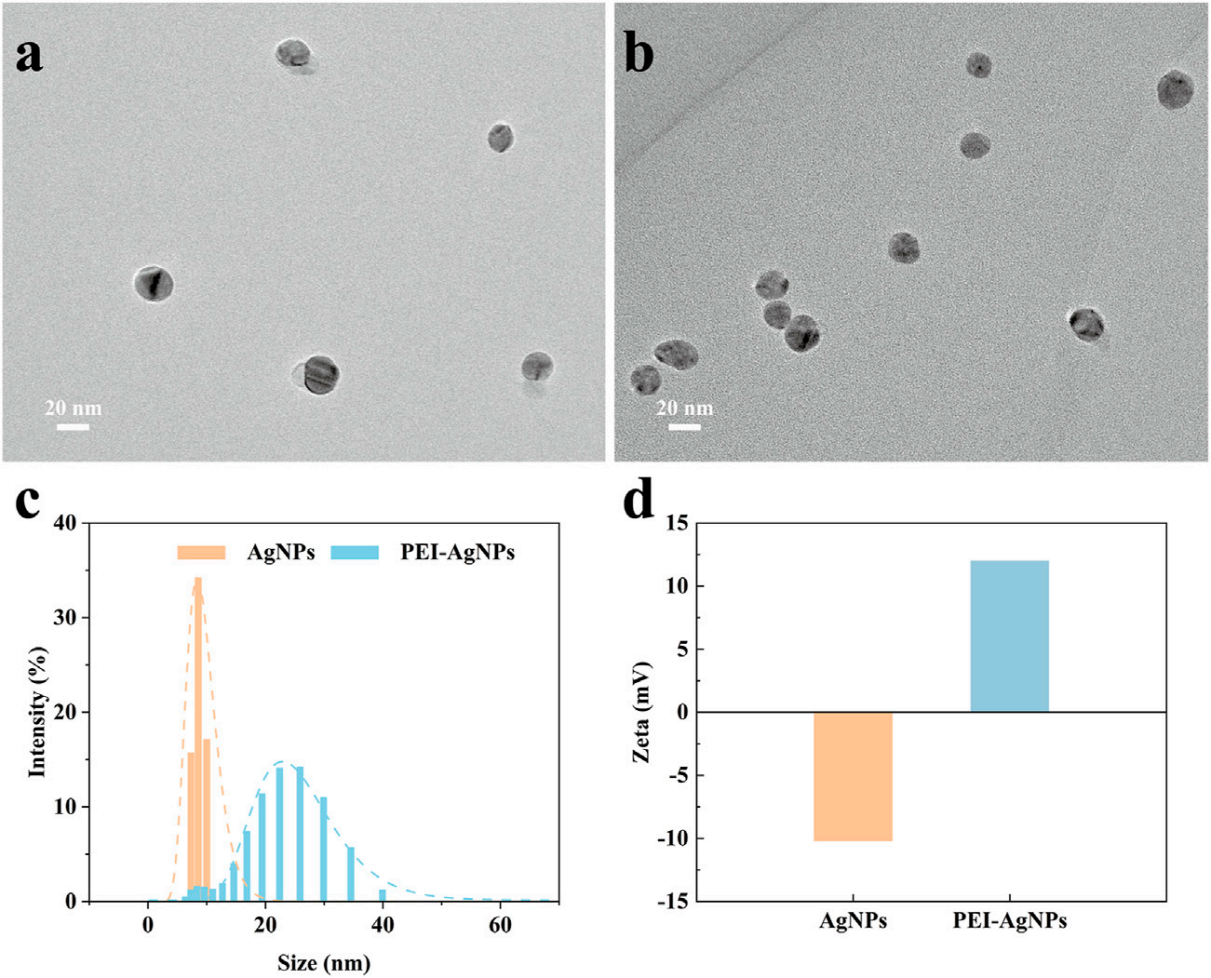
Characterization of unmodified AgNPs and PEI-AgNPs. TEM image of unmodified AgNPs **(A)** and PEI-AgNPs **(B)**. DLS analysis **(C)** and Zeta potential **(D)** of unmodified AgNPs and PEI-AgNPs.

**FIGURE 3:**
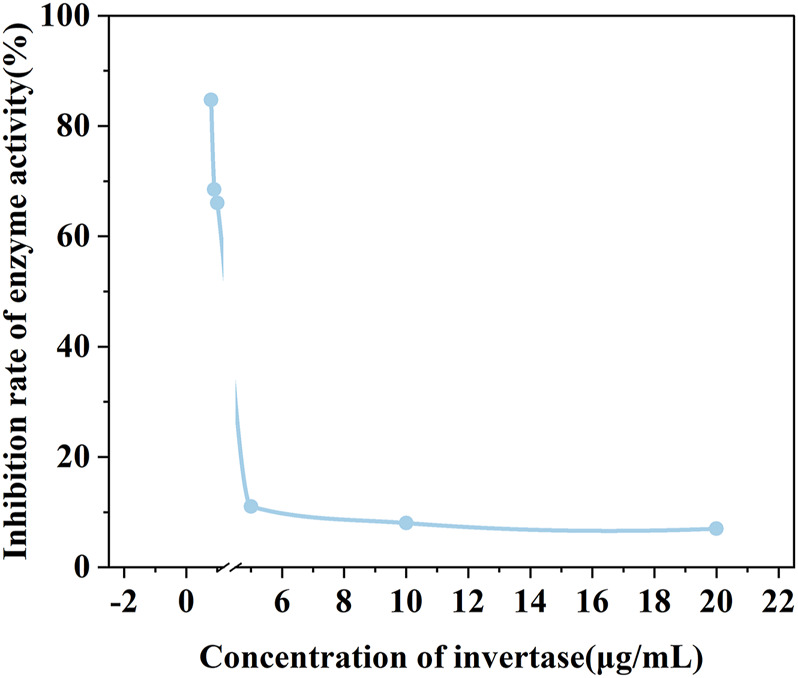
The enzyme activity of AgNPs-invertase complexes with different concentrations of invertase.

Before the feasibility study of our biosensor, PEI-AgNPs were well synthesized according to the previously reported research (Singh et al., 2019). As shown in Figure 2, TEM images of unmodified AgNPs (Figure 2A) and PEI-AgNPs (Figure 2B) were well dispersed with similar sizes. However, after modification with PEI, the obtained PEI-AgNPs showed a larger radius of hydration than unmodified AgNPs according to the DLS analysis (Figure 2C). And as shown in Figure 2D, the Zeta potential changed from −10.2 mV (unmodified AgNPs) to +12 mV (PEI-AgNPs). The results indicated the successful synthesis of positive charged PEI-AgNPs and the synthesis condition was optimized by changing the concentration of PEI (Supplementary Figure S1). And the DLS and zeta potential change of PEI-AgNPs and AgNPs-invertase complexes indicate the success of AgNPs-invertase complexes (Supplementary Figure S2). Next, we optimized the concentration of invertase to form AgNPs-invertase complexes to inhibit the enzyme activity. When the concentration of AgNPs was fixed, the inhibition rate of enzyme activity deserves to decrease with the concentration of invertase increasing. As shown in Figure 3, the inhibition rate reaches the highest when the concentration of invertase is 0.8 μg/ml. Theoretically, the inhibition rate should continue to increase as the concentration of invertase is below 0.8 μg/ml. However, the glucose concentration could not be detected by the PGM when the concentration of invertase is less than 0.8 μg/ml within 20 min. Thus, 0.8 μg/ml of invertase was used to form AgNPs-invertase complexes for the biosensor.

**FIGURE 4:**
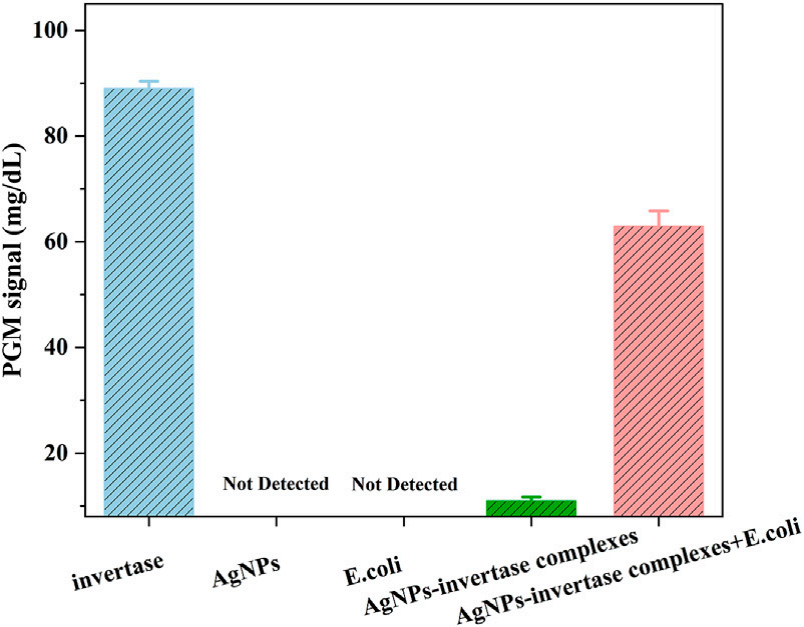
PGM signals under different conditions: invertase, AgNPs, *E. coli*, AgNPs-invertase complexes, and AgNPs-invertase complexes + *E. coli*.

To prove the feasibility of the biosensor, the glucose signal generated under different conditions was measured using a PGM. As shown in Figure 4, the PGM signal of the optimized AgNPs-invertase complexes is significantly lower than that of free invertase, proving the inhibition of enzyme activity by PEI-AgNPs. And the PGM signal is recovered when *E. coli* (10^7^ cfu/ml), as a model of bacteria, was added into the AgNPs-invertase complexes. Obviously, no signal was detected using AgNPs and *E. coli* alone, indicating that the AgNPs and bacteria could not catalyze the hydrolysis of sucrose into glucose. Thus, the PGM signal generated from the mixture of AgNPs-invertase complexes and *E. coli* should be attributed to the activity recovery of the invertase displaced by bacteria from AgNPs-invertase complexes. Therefore, the above results successfully demonstrated the feasibility of the proposed strategy for bacteria detection using nanoparticle-enzyme complexes and the PGM.

### Optimization of Assay Conditions

**FIGURE 5:**
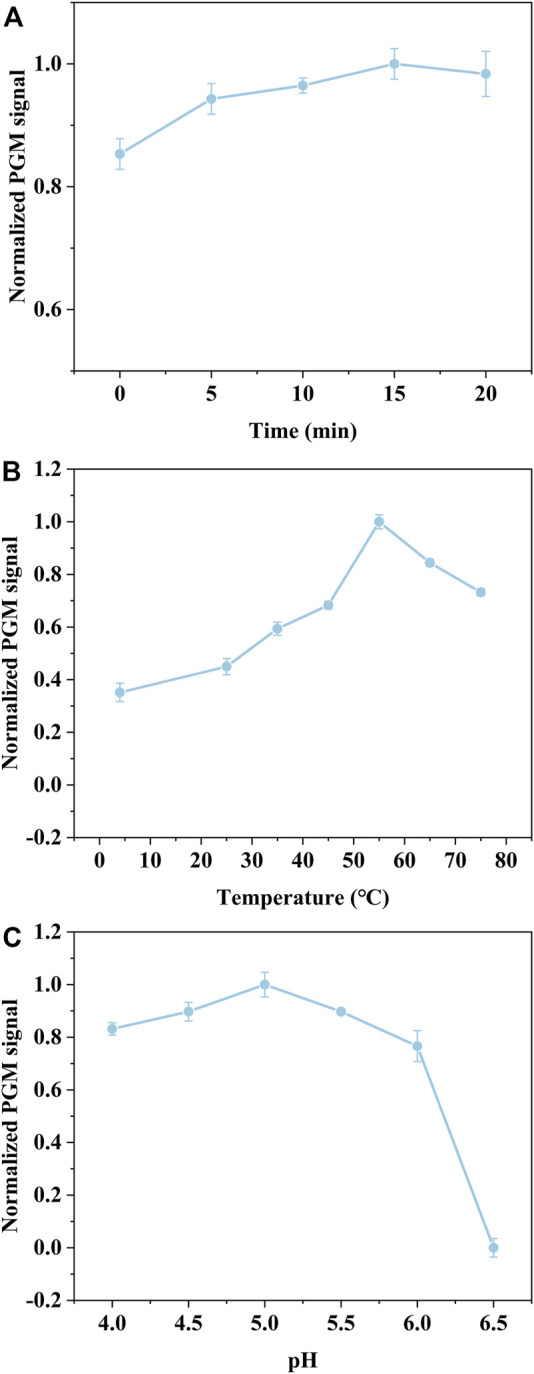
Effect of the reaction time of bacteria combined with AgNPs-invertase complexes **(A)**. Effect of temperature **(B)** and pH **(C)** for the catalytic activity of invertase.

To determine the optimal assay conditions of the proposed detection method, three assay parameters including the reaction time of bacteria combined with AgNPs-invertase complexes, the temperature, and pH for the catalytic activity of invertase, were investigated, since the assay performance is mostly dependent on the catalyst activity of invertase displaced by bacteria from AgNPs-invertase complexes. As the reaction time of bacteria combined with AgNPs-invertase complexes increases, the invertase released into the solution from AgNPs-invertase complexes rises, inducing that the PGM signal increases gradually and reaches a plateau after about 15 min as shown in Figure 5A. Since the catalytic activity and stability of invertase are greatly influenced by temperature and pH, the temperature and pH of the solution were investigated indicating the optimal temperature and pH should be 55°C and 5.0 (Figures 5B,C). Thus, the reaction time of 15 min for bacteria combined with AgNPs-invertase complexes, the temperature of 55°C, and pH at 5.0 was used as the optimal assay conditions for the bacteria detection procedure.

### Detection Performances

**FIGURE 6:**
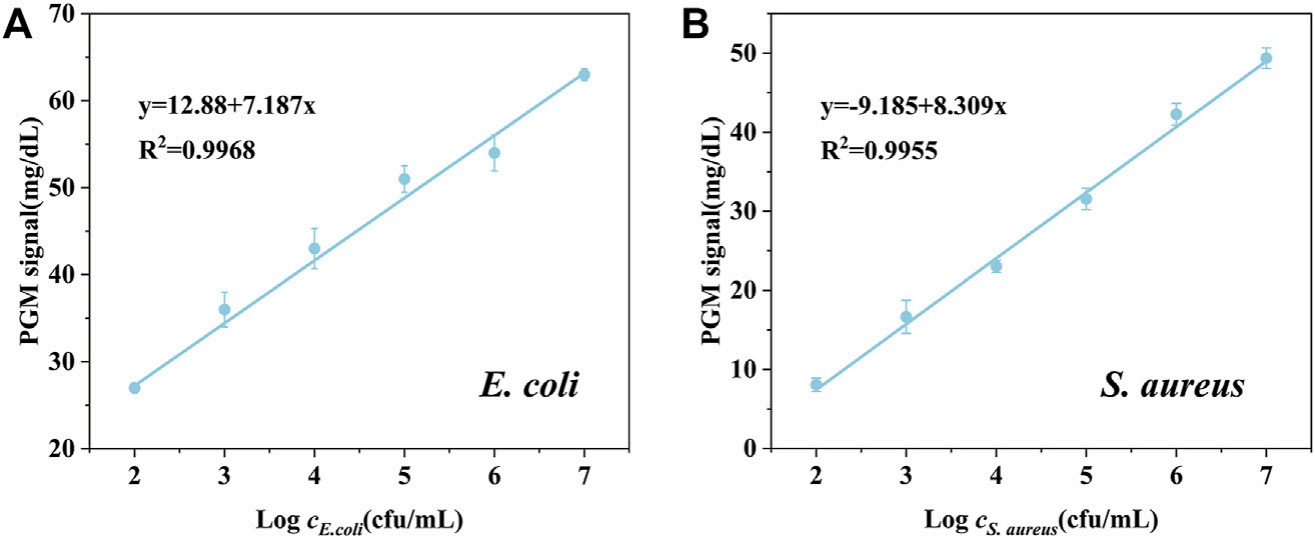
PGM signal against the different concentrations of E. coli **(A)** and S. aureus **(B)**.

Under the optimal assay conditions, we established a universal bacteria detection method using nanoparticle-enzyme complexes and the PGM without multiple washing and rinsing steps. To evaluate the detection performance of our proposed method, *E. coli* and *S. aureus* were selected as the model of Gram-negative bacteria and Gram-positive bacteria. After 15 min of the reaction of bacteria and the AgNPs-invertase complexes, sucrose, the catalytic substrate, was added followed by the measurement of the glucose signal using a PGM. The detection procedure is simple and portable without multiple washing and rinsing steps. There was a good linear relationship between PGM signal and concentration of *E. coli* (from 1.00 × 10^2^ cfu/ml to 1.00 × 10^7^ cfu/ml) or *S. aureus* (from 1.00 × 10^3^ cfu/ml to 1.00 × 10^7^ cfu/ml) (Figure 6). And the limit of detection (3σ/S) of *E. coli* and *S. aureus* are 3 cfu/ml and 7.59 × 10^2^ cfu/ml respectively. The difference in detection sensitivity between *E. coli* and *S. aureus* may be attributed to the variety of sizes and structures of bacteria surfaces.

### Real Sample Detection

To further evaluate the possibility of our proposed detection method of bacteria in real samples, the recovery test was studied by detecting the *E. coli* and *S. aureus* spiked with different concentrations in tap water. As shown in Table 1, the proposed method shows satisfactory recovery values ranging from 93.93 to 106.45%, with RSD values all below 2.52%, indicating the possibility of the nanoparticle-enzyme complexes and the PGM-based bacteria detection method for practical applications. However, there are many different species of bacteria in real sample, which may limit its application for detection of specific bacteria.

**TABLE 1:** Recovery tests of *E. coli* and *S. aureus* in tap water (*n* = 3).

	Bacteria spiked (cfu/ml)	Bacateria found (cfu/ml)	RSD (%)	Recovery (%)
*E. coli*	1×10^3^	0.95×10^3^	0.58	94.73
	1×10^4^	1.04×10^4^	1.53	104.00
	1×10^5^	1.06×10^5^	1.15	106.45
*S. aureus*	1×10^3^	1.13×10^3^	2.52	113.33
	1×10^4^	0.95×10^4^	1.15	95.00
	1×10^5^	0.94×10^5^	1.53	93.93

### AST of Bacteria

**FIGURE 7:**
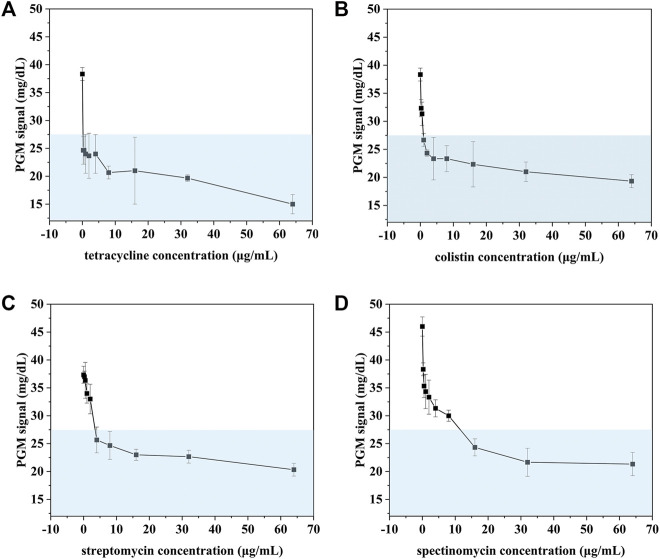
AST of *E. coli* treated with tetracycline **(A)**, colistin **(B)**, streptomycin **(C)**, spectinomycin **(D)** detected using our proposed method.

To investigate the possibility of our proposed method for rapid AST, *E. coli* was chosen as a target and treated with four antibiotics (colistin, spectinomycin, streptomycin, tetracycline). After incubation with different concentrations of antibiotics for 5 h, the concentration of *E. coli* was measured with our proposed method. As shown in Figure 7, the PGM signal decreased with the increase of the concentration of four antibiotics. When the PGM signal is below 27.5 mg/dl, the corresponding concentration of the antibiotic is consistent with the results of MIC testing (Table 2). However, conventional MIC testing needs 18–24 h, which results in irrational empiric therapy and the development of bacterial drug resistance. Our proposed biosensor was demonstrated to be a rapid and reliable method for AST.

**TABLE 2:** The result of AST of *E. coli* with our proposed method and MIC.

Antibiotics	Tetracycline	Streptomycin	Colistin	Spectinomycin
Testing results (μg ml^−1^)	<0.25	4	2	>16
MIC (μg ml^−1^)	<0.25	2–4	2–4	>16

## Discussion

Antibiotic resistance is becoming one of the major challenges for global health (D’Costa et al., 2011; Yelin and Kishony, 2018). The number of people who died from infections with antibiotic-resistant pathogens will surpass cancer in 2050 if no effective measures are taken to reduce misuse and abuse of antibiotics (O’Neil, 2014). Thus, there is an urgent need for rapid bacteria detection and AST of pathogens to treat infected patients with correct antibiotics and reduce the production of antibiotic-resistant bacteria. Current culture-based AST methods usually take at least 1–2 days to get a reliable readout, which makes it difficult to choose suitable treatment in the early, often critical, stages of infection (Syal et al., 2017). As a consequence, the physician often prescribes broad-spectrum antibiotics, which increased the risk of bacterial resistance (Baltekin et al., 2017).

To reduce the time of AST, emerging rapid AST technologies have been developed based on fast phenotypic responses to antibiotics using flow cytometry (Gauthier et al., 2002), Roman (Yang et al., 2019), and mass spectrometry (Yi et al., 2019), or genotypes of bacteria using real-time PCR or whole-genome sequencing (Behera et al., 2019). However, the requirement of expensive instruments and experienced operators limits their application, especially in resource-limited areas. Recently, various biosensors using antibodies (Li et al., 2014) and aptamers (Jo et al., 2018) have been developed for rapid AST by monitoring bacterial growth, which is economical, convenient, and rapid. The use of antibodies and aptamers provided high selectivity, but also complexity for different pathogens through changing corresponding antibodies and aptamers. Thus, a universal bacteria detection platform is needed for the development of a rapid and easy-to-use AST method in medically underserved areas.

Herein, we proposed a universal bacteria detection method for a wide range of bacterial strains based on AgNPs-invertase complexes and PGMs using the electrostatic interactions between AgNPs and bacterial surface, unlike specific recognition elements (e.g., antibody, aptamer). The enzyme-nanoparticle complex-based bacteria colorimetric detection method was first reported by (Miranda et al., 2011) using β-Gal-AuNPs conjugate. The enzyme activity of β-Gal was inhibited by forming β-Gal-AuNPs complexes through strong electrostatic interactions. The presence of bacteria would release β-Gal from β-Gal-AuNPs complexes through competing reactions, resulting in the recovery of the enzymatic activity to catalyze CPRG into a red product. The enzyme activity of lipase and urease could be inhibited by forming lipase-AuNPs (Duncan et al., 2017) and urease-AgNPs complexes (Singh et al., 2019) through strong electrostatic interactions using positively charged nanoparticles and be released by bacteria. However, these methods are rather qualitative, or advanced instruments-required.

Recently, PGM-based biosensors were proved to be a useful tool for universal targets detection with advantages of portability, low cost, simple operation, and ease of getting. Therefore, we developed a novel AgNPs-invertase based biosensor with a PGM for bacteria detection. The invertase released from AgNPs-invertase complexes by bacteria through competing reaction could convert sucrose into glucose, measured using a PGM. Under the optimal assay conditions, there was a good linear relationship between the PGM signal and the concentration of bacteria. Furthermore, our proposed biosensor was proved to be a rapid and reliable method for AST within 4 h with consistent results of MIC testing. The rapid and non-specific detection method provides the opportunity as a universal AST method for various bacteria by monitoring the bacteria concentration treated with antibiotics. And the use of PGMs makes it a portable and convenient method to treat infected patients with correct antibiotics and reduce the production of antibiotic-resistant bacteria, especially for resource-limiting settings.

In summary, the proposed AgNPs-invertase complexes and PGMs based bacteria detection method was proved to be a rapid, portable, and universal detection method with high sensitivity and satisfied recoveries. However, the proposed method could respond to different bacteria without high selectivity which may limit its application for specific bacteria detection in real samples. The use of antibody-coated cationic nanoparticles may resolve the problem (Singh et al., 2019). Surprisingly, the result of four antibiotics towards *E. coli* are consistent with the results of MIC testing, indicating our proposed biosensor to be a rapid and reliable method for AST. Moreover, the nanoparticle-enzyme complexes-based biosensor using other surface-functionalized nanoparticles, enzymes, and portable devices could develop novel point-of-care testing methods and broaden the application towards a variety of analytes.

## Data Availability

The original contributions presented in the study are included in the article/Supplementary Material, further inquiries can be directed to the corresponding authors.
