# Supplementary material for: Rapid Detection and Antimicrobial Susceptibility Testing of Pathogens Using AgNPs-Invertase Complexes and the Personal Glucose Meter
Source: Front Bioeng Biotechnol. 2022 Jan 18;9:795415. doi: 10.3389/fbioe.2021.795415 (PMC8804100; doi:10.3389/fbioe.2021.795415)
Supplement: Supplementary file 1 [file DataSheet1.DOCX]

Supplementary Material





**Supplementary Figure 1.** The absorbance at 410 nm of PEI-AgNPs was synthesized with different concentrations of PEI.


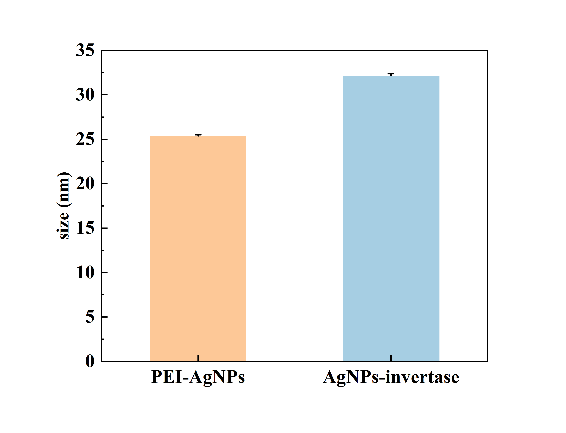


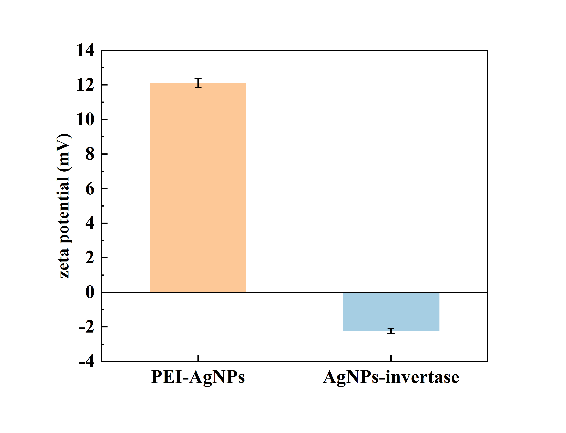


**Supplementary Figure 2.** DLS and zeta potential of PEI-AgNPs and AgNPs-invertase complexes.
